# Cerebellar non-invasive stimulation of social and emotional mentalizing: A meta-analysis

**DOI:** 10.1162/imag_a_00334

**Published:** 2024-10-28

**Authors:** Frank Van Overwalle, Naem Haihambo, Qianying Ma, Meijia Li, Rocío Martínez-Regueiro, Ines Argoub, Mahyar Firouzi, Natacha Deroost, Chris Baeken, Kris Baetens

**Affiliations:** Faculty of Psychology and Center for Neuroscience, Vrije Universiteit Brussel, Brussels, Belgium; Language Pathology and Brain Science MEG Lab, School of Communication Sciences, Beijing Language and Culture University, Beijing, China; NeuCogA-Aging Group GI-1807-USC, Department of Clinical Psychology and Psychobiology, Faculty of Psychology, Universidade de Santiago de Compostela, Galicia, Spain; Rehabilitation Research Group, Department of Physiotherapy, Human Physiology and Anatomy, Vrije Universiteit Brussel, Jette, Belgium; Department of Psychiatry, Vrije Universiteit Brussel (VUB), Universitair Ziekenhuis Brussel (UZ Brussel), Brussels, Belgium; Ghent Experimental Psychiatry (GHEP) Laboratory, Department of Head and Skin, Ghent University, Department of Psychiatry, Ghent University Hospital, Ghent, Belgium; Department of Electrical Engineering, Eindhoven University of Technology, Eindhoven, The Netherlands

**Keywords:** non-invasive cerebellar stimulation, TMS, tDCS, social mentalizing, theory of mind, social sequencing, emotion

## Abstract

The present meta-analysis investigated the impact of non-invasive stimulation, using transcranial direct current stimulation (tDCS) and transcranial magnetic stimulation (TMS) targeting the posterior cerebellum, on social and emotional mentalizing about others. Prior research has convincingly shown that the posterior cerebellum supports social and emotional cognition. We identified 14 studies targeting the cerebellum with appropriate control conditions (i.e., sham, control site), which exclude general learning effects of the task or placebo effects. The studies included 29 task conditions where stimulation before or during a social or emotional task was applied on healthy samples. The results showed significant evidence that sustained anodal tDCS and TMS generally improved social and emotional performance after stimulation, in comparison with sham or control conditions, with a small effect size. In contrast, cathodal stimulation showed mixed facilitatory and inhibitory results. In addition, short TMS pulses, administered with the aim of interfering with ongoing social or emotional processes, induced a small but consistent inhibitory effect. Control tasks without social or emotional components also showed significant improvement after sustained anodal tDCS and TMS, suggesting that transcranial stimulation of the cerebellum may also improve other functions. This was not the case for short TMS pulses, which did not modulate non-social and non-emotional control tasks. Taken together, this meta-analysis shows that cerebellar neurostimulation confirms a causal role of the cerebellum in socio-emotional cognition, has a small but significant effect on improving socio-emotional skills, and may, therefore, have important clinical applications in pathologies where social and emotional cognition is impaired.

## 1 Introduction

While the critical involvement of the cerebellum in movement and motor-related tasks is well established, it is increasingly recognized that the cerebellum also has important functions in the non-motor domain, such as cognition and emotion. The role of the cerebellum in social cognition and social behavior has only recently been discovered in an influential large-scale meta-analysis (Van Overwalle et al., 2014,^2015^). Social and emotional behavior permeates a large part of human life. This is true not only of direct face-to-face interpersonal interaction and communication at work, in the family and during leisure time, but also of most interactions on social media. It is also reflected in various art forms such as literature, theater, film, music, and opera that delve into human mental and emotional states that motivate actions.

In complex human interaction, social cognition and behavior is supported by the capacity to read the mental and emotional states of the self and other people, which is termed*mentalizing*. Examples of a person’s mental states are intentions, thoughts, emotions, desires, traits, and so on. This capacity is subserved by the*mentalizing*brain network (Schurz et al., 2014;Van Overwalle, 2009), which encompasses a large part of the*default mode*network (Andrews-Hanna et al., 2010,^2014^). While social behavior and emotional experiences are initially driven by more low-level circuits such as the*action observation (or mirror)*network involved in the observation of goal-directed human movement (Molenberghs et al., 2012;Van Overwalle & Baetens, 2009), and the*limbic*system involved in core emotional sensations (Lindquist et al., 2012), ultimately these early signals are further processed and modulated by a high-level mentalizing network in the neocortical cerebrum (Schurz et al., 2021) and posterior cerebellum (Van Overwalle et al., 2020).

Neuroimaging research on the social and emotional functionality of the cerebellum is growing and has uncovered a complex functional anatomy of the cerebellum (Buckner et al., 2011;Ji et al., 2019;King et al., 2019;Van Overwalle et al., 2023). However, the observational methodologies (e.g., fMRI) used in these studies tend to only provide correlational evidence on the functionality of the cerebellum, with little information on its causal impact. To investigate causality, researchers often apply non-invasive neurostimulation and measure its impact on brain functioning, plasticity, and behavior. This is also a growing approach in cerebellar research, particularly due to the fact that the posterior cerebellum is located close to the base of the human skull, making it easily accessible for non-invasive stimulation. However, precise targeting of stimulation within the cerebellum is challenging due to its highly convoluted (i.e., folded) structure. Non-invasive cerebellar stimulation has the added advantage of providing potential directions for therapeutic interventions for improving social and emotional cognition, especially in populations with social difficulties, such as autism spectrum disorder (American Psychiatric Association, 2013).

Non-invasive techniques that have recently been investigated intensely in the socio-emotional domain are transcranial magnetic stimulation (TMS) and transcranial direct current stimulation (tDCS). A cursory review of several meta-analyses on transcranial stimulation of social and emotional processes reveals that most studies do not stimulate the cerebellum (Bell & DeWall, 2018;Christian & Soutschek, 2022;Smits et al., 2020;Yang et al., 2018;Yuan et al., 2022). One meta-analysis did focus on the cerebellum (Oldrati & Schutter, 2018), but included a large variety of motor and non-motor tasks, and only included studies that applied tDCS. Therefore, the aim of the present meta-analysis is to provide an extensive statistical analysis of transcranial stimulation on social and emotional processes, in which tDCS and TMS target the cerebellum. Other non-invasive stimulation techniques modulating socio-emotional mentalizing were not found in the literature, except one study applying transcranial random noise stimulation (Malatesta et al., 2024). This study was not included in this meta-analysis, because this is the only study on this novel promising technique we are aware of. To understand how the cerebellum impacts social and emotional cognition, a brief overview of the cerebellar anatomy and functionality, and its connectivity with the cerebrum, is first provided.

### 1.1 Anatomy and function of the cerebellum

The cerebellum has a surprisingly large computational capacity, supported by a surface approximately 80% the size of the cerebral neocortex (Sereno et al., 2020), and contains an astonishing 80% of the neurons in the human brain (Lent et al., 2012). The anterior cerebellum supports motor processes, which are evolutionarily older, while the posterior cerebellum supports social and emotional processes, which are evolutionarily younger (Keren-Happuch et al., 2014;Stoodley & Schmahmann, 2009;Van Overwalle et al., 2014). The socio-emotional functions are located in the bilateral Crus 1 and 2 (part of lobule VII at the most posterior part of the cerebellum), and in the inferior posterior cerebellum (lobule IX, located just below the anterior cerebellum). These socio-emotional areas are critical parts of the cerebellar mentalizing network (seeFig. 1).

**Fig. 1.**
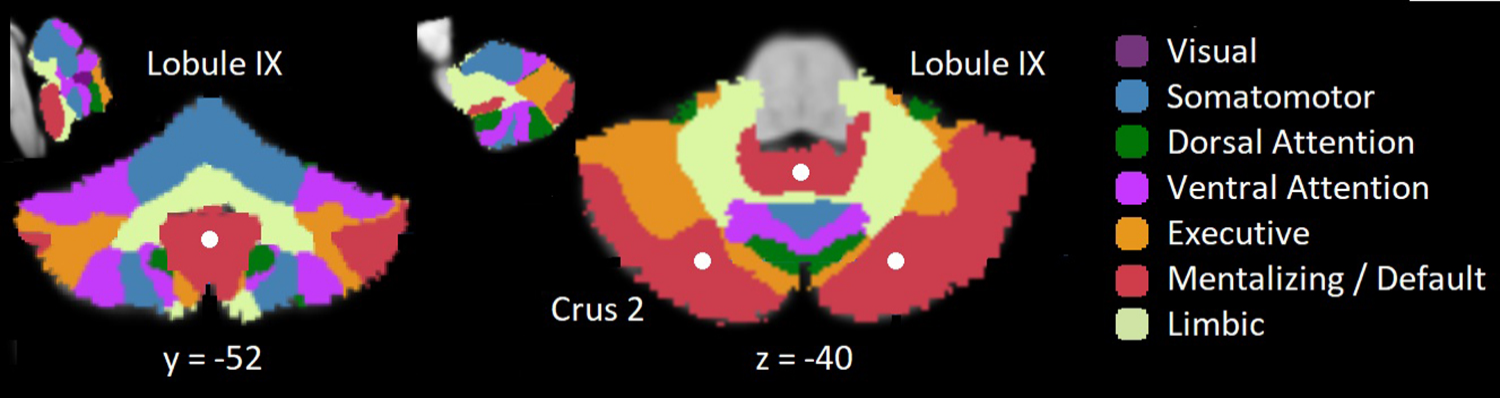
View of the cerebellum from the back [Left] and top [Middle] (at MNI coordinates y = -52 and*z*= -40, respectively), showing the center of the key areas in Crus 2 and Lobule IX (white circles) of the mentalizing network (red). Color legend of all networks byBuckner et al. (2011)[Right]. The anterior–posterior insets (top left and top middle) illustrate that lobule IX and Crus 2 (at MNI coordinates = 0 and 14, respectively) are easily accessible for non-invasive neurostimulation by targeting the posterior scalp, but that other brain networks are also impacted. MNI coordinates of the centers are Crus 2: x = ±25, y = -75,*z*= -40, and Lobule IX: x = 0, y = -52,*z*= -40.

Importantly, the cerebellum forms reciprocal connections with the cerebral neocortex through multiple synapses; downward through the pons and upward through the thalamus (Biswas et al., 2019;Granziera et al., 2009;Kelly & Strick, 2003;Na et al., 2019;Wagner et al., 2019;Zhu et al., 2023). The reciprocal structure of the connections is essential, because it implies that neural signals are exchanged between specific areas of the same networks in both brain structures, so that the cerebellum receives input from, and sends feedback back to the same distant functional areas in the neocortex. The topographical arrangement of these reciprocal connections within the cerebellum determines its different functionalities and contributes to its multifaceted functional network structure (Buckner et al., 2011;Ji et al., 2019;Van Overwalle et al., 2023). This includes the mentalizing network, depicted inFigure 1, as well as major networks that have also been identified in the cerebrum.

The uniform neural architecture across the whole cerebellar cortex has generated a variety of speculations on an underlying, general function of the cerebellum, including the idea that the cerebellum acts as a biological clock, a timing machine, and error detector, and is involved in automatization, prediction, and preparation (Schmahmann et al., 2019). An attractive theory in a prediction framework that integrates many of these functions is the “*sequence detection hypothesis*” put forward byLeggio and Molinari (2015)and supported by earlier work (Ito, 2008). In brief, according to this hypothesis, the cerebellum identifies the temporal sequences of motor and mental actions such as low-level sensory consequences of one’s intended movements or higher-level inferences of social and emotional actions, and encodes these in internal cerebellar models. This allows the cerebellum to automatize and fine tune a variety of motor and cognitive processes and its sensory or mental consequences, which enables fast feedback on errors when the sequence of events does not match internal expectations. These error signals are sent to the cerebrum and help to quickly adjust ongoing motor and mental behaviors within the context, leading to more appropriate social and emotional actions.

A series of recent studies have confirmed the critical role of the cerebellum in identifying sequencing of social actions, by consistently showing stronger posterior cerebellar activation when dynamic sequences of social actions were involved versus no sequences (for reviews, seeVan Overwalle & Heleven, 2023;Van Overwalle et al., 2021). Several studies also compared dynamic versus static facial emotional expressions, but failed to report consistent differences in cerebellar activity (see meta-analyses byLiu et al., 2021;Zinchenko et al., 2018). In contrast, a recent monkey study demonstrated stronger cerebellar activation in dynamic versus static facial emotional processing (Dureux et al., 2023). Moreover, a non-invasive cerebellar stimulation study using transcranial random noise, mentioned earlier (Malatesta et al., 2024), found reduced accuracy to discriminate static sad facial expressions, but increased accuracy to discriminate dynamic sad facial expressions; no effects emerged with happy faces. Given the key role of the cerebellum in processing sequences, it seems plausible to assume that by using dynamic rather than static input of social and emotional stimuli, non-invasive cerebellar stimulation might be more effective or its effects more easily detected.

### 1.2 Non-invasive cerebellar stimulation

Because the cerebellum is increasingly recognized as supporting social and emotional cognition, it has become a target for non-invasive neurostimulation involving tDCS and TMS. As noted earlier, the position of the mentalizing area in Crus 1 and 2 at the back of the cerebellum makes it an easy target for non-invasive stimulation via the scalp (Fig. 1). Generally, both tDCS and TMS lead to functional modulations in brain cortices. The focality of stimulation is relatively wide for standard tDCS and narrow for TMS. However, this is highly dependent on the type and size of tDCS electrodes and TMS coil. For instance, conventional tDCS typically uses two rather large electrodes, often resulting in a diffuse electric field with low spatial focality (Masina et al., 2021), whereas novel high-definition (HD) tDCS montages can stimulate with higher specificity by employing multiple, and often smaller electrodes.

tDCS does not directly induce action potentials, but rather changes brain excitability by modulation of the thresholds for stimulation by other neurons to elicit an action potential (Cattaneo et al., 2022;Stagg et al., 2018;van Dun et al., 2017). It is generally found that positively charged anodal tDCS results in enhancement of cortical excitability, while negatively charged cathodal tDCS results in mixed effects, causing enhancement or inhibition (e.g., after 20 min of 2 mA vs. 1 mA, respectively;Batsikadze et al., 2013). This polarity effect is not very robust for tDCS on the cerebellum either, leading to either enhancement or inhibition (Klaus & Schutter, 2022;van Dun et al., 2017). tDCS is conventionally applied at an intensity of 1–2 mA for 20 min. However, tDCS can have non-linear effects, so that stimulation which lasts too long (≥25 min) or is too intense (≥2 mA) may decrease or reverse conventional tDCS effects (e.g.,Stagg et al., 2018). Note that tDCS is a net effect over many thousands of neurons, which respond in different or even opposite directions depending on their orientation in relation to the induced electric field and stimulation electrode type, intensity, and duration (Stagg et al., 2018).

TMS can directly induce an electric current in the underlying brain area which leads to neuronal depolarization and an action potential. TMS can modulate cortical excitability by providing short pulses while participants are engaged during a task, or by providing repetitive TMS (a long series of pulses) over a period of 10 to 20 min before a task. At low frequencies (below 1 Hz), repetitive TMS results in the suppression of cortical excitability, while at higher frequencies (above 5 Hz), repetitive TMS mostly increases excitability (Barahona-Corrêa et al., 2018). TMS is a net effect of action potentials of many neurons traveling along axons in different directions, which depend on the underlying cortical anatomy, coil type, and placement, and stimulation intensity, duration, and frequency (Siebner et al., 2022).

Both tDCS and repetitive TMS can have long-term after effects which are thought to be mediated by neurotransmitter levels such as the relative concentration of inhibitory gamma-aminobutyric acid (GABA) versus excitatory glutamate, which determine changes in synaptic strength (Ferrucci et al., 2023;Hameed et al., 2017;Heimrath et al., 2020;Nwaroh et al., 2020;Siebner et al., 2022;Stagg et al., 2018). It is believed that anodal tDCS and high-frequency repetitive TMS tend to decrease GABA levels and increase glutamate levels, promoting excitatory effects, while cathodal tDCS and low-frequency rTMS can reverse neurotransmitter levels, resulting in inhibitory effects (Ferrucci et al., 2023;Hameed et al., 2017). This change in balance between excitation and inhibition in the brain can influence various motor and cognitive functions. The most common approach in non-invasive stimulation is sustained stimulation with tDCS or repetitive TMS for 10 to 20 min to specific regions, and measuring afterward how this modulates task performance. This approach also has appealing clinical implications for use as a therapeutic intervention, although clinical effectiveness typically requires multiple sessions. Such multi-session studies were not included in this meta-analysis because they did not comply with the inclusion criterion of involving objective social or emotional performance measures (cf. accuracy or response times). Typically, task performance after active stimulation is compared with sham stimulation. Sham tDCS is usually delivered to mimic the sensation of stimulation by gradually ramping up stimulation and then gradually down; this is done at the start and at the end of the session, without providing any active stimulation in between.

An example of this sustained modulation methodology targeting the cerebellum is the tDCS study byOldrati et al. (2021). The researchers showed participants video clips depicting persons performing various social actions (e.g., grasping an apple) that ended with a clear purpose that was either individual (i.e., to eat themselves) or interpersonal (i.e., to offer to another person), while contextual information in the video (i.e., placemat on which the offer was made) allowed to predict the persons’ purpose to some degree. Compared with sham stimulation, anodal tDCS (1.5 mA for 20 min) targeting the vermis increased participants’ ability to predict the correct purpose, whereas cathodal tDCS impaired it. Critically, tDCS did not impact non-social prediction (involving physical shapes). Another example is a TMS study byHeleven et al. (2021). The researchers applied low-frequency repetitive TMS (1 Hz for ~17 min) over the cerebellar vermis before participants performed two social sequencing tasks: Picture and Verbal Sequencing. Participants had to generate the correct chronological order of various stories presented randomly in cartoon-like pictures or short sentences that required mentalizing (i.e., true and false beliefs, social scripts) as well as mechanical (i.e., physical) control stories. Stimulation resulted in faster responses from pre- to post-stimulation for all types of story sequence after TMS, whereas almost no effects were observed after sham stimulation.

Another stimulation approach is to acutely disrupt neural activity by delivering brief TMS pulses during or after (e.g., 100 ms or more) stimulus presentation, to observe how this interferes with task performance. Sham TMS is typically provided by delivering no magnetic pulses or providing TMS at a control site. As an example of this methodology,Ferrari et al. (2023)presented one of two different emotional facial expressions (i.e., happiness or anger/fear) and applied a short triple-pulse TMS of 20 Hz targeting the left cerebellum before the second face presentation. This manipulation decreased accuracy in discriminating the emotional facial expressions in comparison with a control TMS over the vertex.

The application of non-invasive stimulation targeting the human cerebellum introduces new opportunities to study its role in social and emotional functioning. However, given that this research is still in its infancy, little is known about the magnitude of its effect. A recent scoping review (Ciricugno, Oldrati, et al., 2024) offered an overview of research on cerebellar stimulation for the modulation of socio-emotional functions during the last two decades, and concluded that cerebellar neurostimulation is effective in improving social abilities in healthy individuals and reducing social and affective symptoms in different neurological and psychiatric populations associated with cerebellar damage or impairments. However, this analysis had some limitations, which were avoided in the present meta-analysis. First, no statistical measures were provided to evaluate the magnitude of the effects. Second, many studies did not include appropriate control conditions (e.g., sham, control sites) so that they cannot exclude a facilitatory role of general learning of the task or placebo effect. Third, measures in many studies included only clinical symptoms and did not measure social or emotional functions.

The present meta-analysis is stricter in terms of methodology and the focus of the studies. First, we required at minimum an adequate control condition, which clearly excluded increasing familiarity with the task or placebo effects as potential confounding factors for improved performance. This control requirement ruled out many studies of clinical populations. Second, we required blinding of participants, but not of the researchers (i.e., none of the studies in this meta-analysis met this last requirement). Third, we focused on tasks that involved perceiving and understanding the social and emotional behavior of others, using objective measures such as task accuracy or response times. This excluded studies that only measured participants’ clinical symptoms. These requirements left us with only one clinical study (De Vidovich et al., 2016). We, therefore, decided to leave out this clinical study and to focus only on studies with neurotypical participants, to keep this meta-analysis more homogeneous.

The included studies involve a wide range of tasks, which can be categorized according to two independent dimensions. A*social-emotional*dimension characterizes the tasks as*social*, going from low-level goal-directed inferences of biological motions (i.e., action observation or “mirror” tasks;Van Overwalle & Baetens (2009)) to higher-level inferences of the mental state of other persons, including their beliefs and traits (i.e., social mentalizing;Schurz et al., 2014;Van Overwalle & Baetens, 2009) versus*emotional*as observed via facial or bodily expressions (i.e., emotional mentalizing;Schurz et al., 2021;Van Overwalle et al., 2020). Some examples of these tasks have been described in the preceding paragraphs (cf. action observation inOldrati et al., 2021; social mentalizing inHeleven et al., 2021; emotional mentalizing inFerrari et al., 2023). Another*static-dynamic*dimension characterizes the task stimuli as either*static*, by showing pictures of facial expressions (in the majority of the short-pulse studies) versus*dynamic*, by showing sequences of human movement (e.g.,Ferrari, Ciricugno, & Cattaneo, 2022;Oldrati et al., 2021), by requiring participants to generate chronological sequences of social actions (e.g.,Haihambo et al., 2024;Heleven et al., 2021) or by implicitly learning a sequence that is covertly repeated (e.g.,Ma et al., 2023). Note that none of the selected studies with emotional stimuli contained dynamic facial or bodily expressions.

By combining social and emotional domains, using static and dynamic stimuli, applying both tDCS and TMS techniques, and using both sustained modulation and short inference approaches, the present analysis might provide an informative database and evidence on the efficacy of non-invasive stimulation on socio-emotional processes. We focus on tasks involving the accurate judgment of social or emotional events using objective measures such as accuracy or reaction times. Although the diversity of tasks and stimuli between studies may provide a broad framework for generalizing the effects of non-invasive neurostimulation in social and emotional cognition, it also introduces a substantial heterogeneity between studies which may potentially weaken the overall effects. This meta-analysis may perhaps be informative on some methodological issues about effective cerebellar protocols, such as the anodal versus cathodal polarity of tDCS, and the stimulation site at the vermal versus lateral cerebellum.

## 2 Method

### 2.1 Study selection

A literature review was performed on November 20, 2023 using PubMed to identify potential studies for the meta-analysis. The search terms used in the “title/abstract” fields were (“cerebellum” or “cerebellar”) and (“transcranial direct current stimulation” or “tDCS” or “transcranial magnetic stimulation” or “TMS” or “transcranial alternating current stimulation” or “tACS” or “non-invasive electrical stimulation” or “transcranial electrical stimulation” or “non-invasive brain stimulation” or “NIBS” or “transcranial brain stimulation” or “non-invasive stimulation” or “transcranial stimulation”) and (“social” or “mentalizing” or “emotion” or “emotional” or “affective”). To ensure no studies were missed, the reference lists of recent reviews on cerebellar non-invasive stimulation (Cattaneo et al., 2022;Ciricugno, Oldrati, et al., 2024;Escelsior et al., 2019;Ferrari, Ciricugno, & Cattaneo, 2022;Gatti et al., 2021;van Dun et al., 2017,^2022^;Wessel et al., 2023) were also examined.

Studies were selected after reading title, abstract or full text, if they met the following criteria:

(a) targeted a position over the cerebellum,
(b) used a task involving the judgment of social or emotional events,
(c) used an objective measure such as accuracy or reaction times as the dependent variable,
(d) used an appropriate control condition to avoid placebo or task learning effects, including a tDCS/TMS sham control, a TMS control site, or a control condition or sample in a pre–post stimulation design,
(e) involved neurotypical adults; excluding clinical samples as well as patients with predominantly motor-related impairments (Maas et al., 2022;Ruggiero et al., 2022) which may interfere with potential effects of non-invasive stimulation due to changes in motor responses (e.g., response times) rather than mental processes, and
(f) were published in a peer-reviewed English language journal.

Studies were excluded after full-text reading (see exemplary references between parentheses) if they

(g) involved simultaneous stimulation of two brain areas making it impossible to identify the source and/or direction of stimulation (Catoira et al., 2023;D’Urso et al., 2021;Newstead et al., 2018),
(h) did not include social or emotional task manipulations or measures (Demirtas-Tatlidede et al., 2011;Ferrari et al., 2021;Garg et al., 2016;Ho et al., 2014;Schutter et al., 2003),
(i) involved a pre–post stimulation design without sham or control condition (neurotypical sample inDemirtas-Tatlidede et al., 2010;De Vidovich et al., 2016) or
(j) single-case reports.

Due to the small number of available studies, study quality was not considered in the decision of inclusion. The selection process is depicted inFigure 2.

**Fig. 2.**
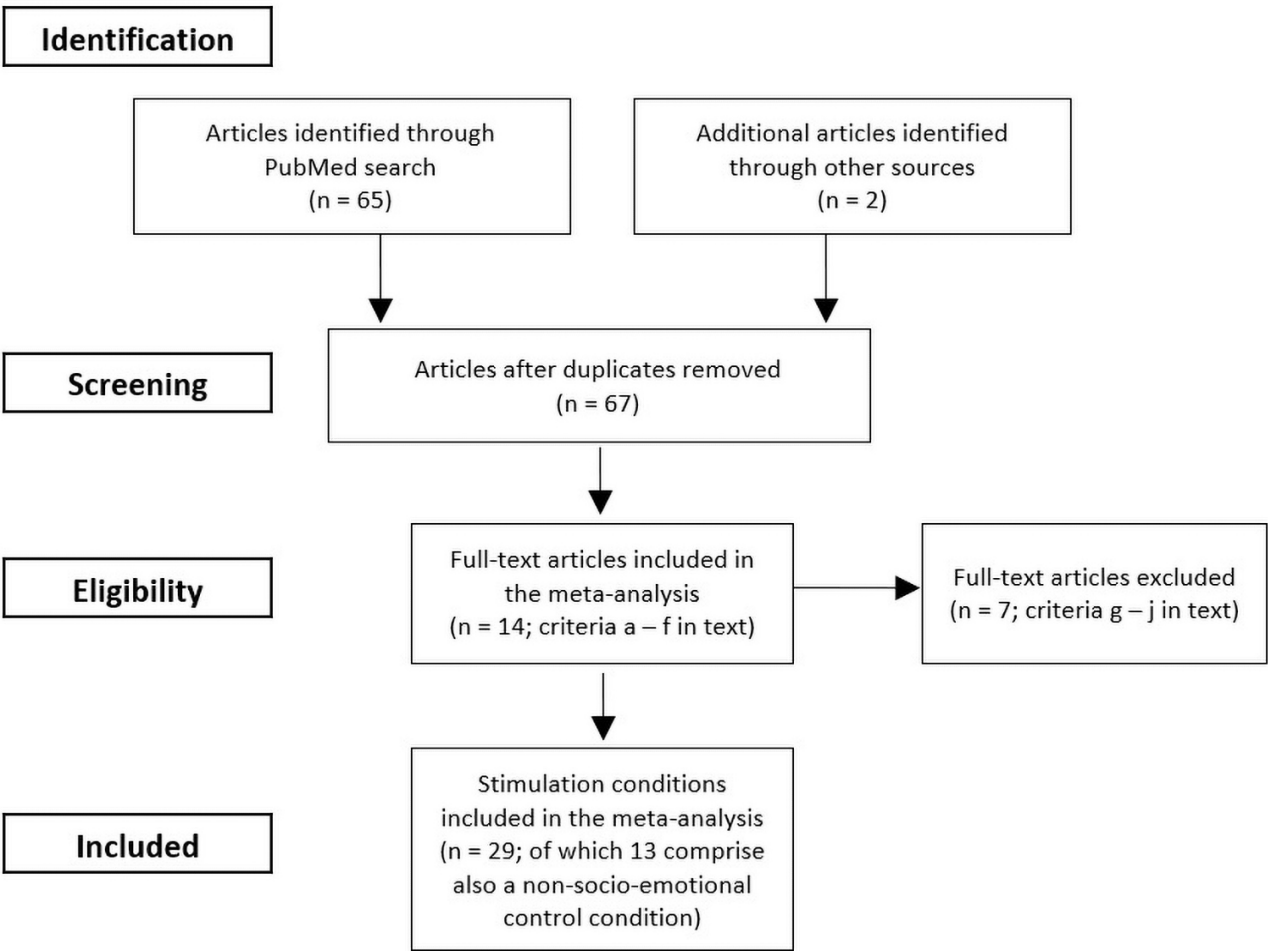
Prisma flowchart of the meta-analysis on non-invasive transcranial stimulation of social and emotional cognition.

The literature search yielded 65 articles, and together with 2 unpublished articles (under review at that time; from our own laboratory or available in bioRxiv) this resulted in 67 articles in total. To make sure that we did not miss studies on clinical populations, we widened the original search by dropping the field restriction for the keywords related to the cerebellum (i.e., all fields were searched), keeping the search terms related to tDCS and TMS, and adding the following terms in the “title/abstract” field: “Obsessive” or “Compulsive” or “OCD” or “Schizophrenia” or “Schizophrenic” or “Schizoid*” or “Borderline” or “Depression” or “Depressive” or “MDD” or “Bipolar” or “Addiction” or “ADD” or “Addictive”. These keywords were based on a review article discussing the impact of the cerebellum on clinical dysfunctions (Van Overwalle et al., 2022). This second search provided 17 additional articles that were not identified in the first search. However, these articles were not suited for the meta-analysis on the basis of our exclusion criteria.

After screening the title and abstract, 22 articles were left for full-text review based on the inclusion criteria. Of these, 7 were further discarded based on one or more exclusion criteria, leaving 14 articles selected for the current meta-analysis. Note that the selected articles included 1 to 4 stimulation studies that together provided 1 to 6 relevant stimulation conditions that satisfied our criteria, resulting in 29 stimulation conditions measuring socio-emotional task performance. In addition, these articles involved 13 stimulation conditions with non-socio-emotional task measures (see Prisma flowchart inFig. 2).

The studies were categorized according to their social or emotional domain, and whether they involved sequences or not, depending on the task stimuli and instructions (see introduction). Two studies with emotional stimuli deserve some clarification. In the study bySchutter and Van Honk (2009), “in the emotion regulation task (ERT), participants were presented with a series of unpleasant highly arousing pictures . . . subjects were instructed to either just experience any feelings elicited by the unpleasant pictures or to neutralize any feelings by cognitive re-appraisal” (pp. 29–30), without any further detailed instructions. The effect of neurostimulation was analyzed for both emotion “experience” and “reappraisal” exposure instructions combined, and no differences between these two instructions were reported (suggesting that the “reappraisal” instruction was ineffective). Given the lack of differences between instructions and because re-appraising (e.g., eschewing from) highly arousing unpleasant pictures might reflect a natural reaction to these stimuli, we decided to include this study. The negative mood after exposure to the highly unpleasant stimuli was increased after rTMS compared with sham, and this was, therefore, taken as a direct effect of the neurostimulation. Note that removing this study would not change much for the overall results of the sustained anodal tDCS and rTMS studies, as the pooled effect size would drop only slightly from 0.20 to 0.17, and would remain significant (*z*= 2.70,*p*< 0.007).

In the study byGamond et al. (2017), participants were primed by pictures of ingroup or outgroup members, immediately followed by positive or negative adjectives and had “to indicate as fast as possible whether the adjective was positive or negative” (p. 933). An emotional ingroup bias was (indirectly) determined by responses that were “significantly faster to positive than negative adjectives when primed by ingroup” (p. 935). Since the primary measure was emotional, the study was categorized as belonging to the emotional domain.

### 2.2 Data extraction

Response times and performance accuracy on social and emotional tasks were the primary dependent outcome variables. If more than one dependent variable or social/emotional task was available, we selected the one with the strongest effect after stimulation for further analysis, as a measure of the maximum potential of a given stimulation methodology and task context, as these typically differ between studies. For the meta-analysis, we used the effect size metric Hedges’*g*. To calculate Hedges’*g*(Borenstein et al., 2009a), we used the same strategy and general procedures as in an earlier meta-analysis (Barahona-Corrêa et al., 2018). We took the following descriptive data from each study: sample size, mean, and standard deviation (SD) of the outcome measure for the active stimulation and the sham/control site stimulation. SD was also computed from the standard error (SE), using the formula SD = SE * √n where n = sample size. When performance was measured across multiple time points or for multiple task outcomes, we calculated the aggregate mean = [(μ_1_+ μ_2_+… + μ_n_) / k] and pooled SD = [√((SD_1_^2^+ SD_2_^2^+… + SD_n_^2^) / k)], where k = total number of data points. If mean and SD were not provided, we mailed the original authors for the original data. We obtained almost all the data from the studies conducted by Chiara Ferrari (Ciricugno et al., 2024;Ferrari, Ciricugno, Battelli, et al., 2022;Ferrari, Ciricugno, Urgesi, et al., 2022;Ferrari et al., 2018,^2023^), for which we are very grateful. Data from older studies were most often not available any more after duly searched by the authors, while other authors did not respond. For the remaining unavailable data, we took the*t*and*F*statistics from direct comparisons between active and sham/control conditions. If the*p*-value was reported but not the*t*-value, the reported*p*-value was converted to the*t*-value using the Excel formula TINV to calculate the inverse of the two-tailed Student’s*t*distribution. If control task conditions were present without social or emotional elements, we extracted the same data if available.

For within-participant studies with dependent samples, additionally a correlation coefficient between pre- and post-treatment scores was extracted. We computed the correlation coefficient using the formula*r*= (SD_pre_^2^+ SD_post_^2^− SD_D_^2^)/(2 * SD_pre_* SD_post_) with SD_D_^2^= (n * (M_post_− M_pre_)^2^)/t^2^, where t^2^is the pre–post paired*t*-test value, M_pre_(SD_pre_) is the pre-stimulation mean score (standard deviation), and M_post_(SD_post_) the post-stimulation mean score (standard deviation;Morris & DeShon, 2002). For 5 out of 28 within-participant conditions with or without socio-emotional tasks (or 18%), the reported or obtained values were insufficient for this computation, and in these cases, we used a default*r*value of 0.80. This value was based on the pre–post correlations which were available for all the other within-participant conditions, which averaged to a*r*= 0.79 and was rounded to 0.80. To analyze how sensitive the obtained results were, we also conducted the analyses with an*r*ranging from 0.70 to 0.90, in steps of 0.05.

### 2.3 Data analysis

To estimate effect sizes, we computed Hedges’*g*for each study, using the Meta-essentials workbooks (https://www.erim.eur.nl/research-support/meta-essentials) for independent samples (between design) and dependent samples (within design). The obtained Hedges’*g*and its standard error were then entered for an analysis across all between and within designs. Individual studies were weighed according to the inverse variance weighting method, with an added between-studies variance component based on the DerSimonian-Laird estimator (Sánchez-Meca & Marín-Martínez, 2008). Confidence intervals were estimated using the weighted variance method (Sánchez-Meca & Marín-Martínez, 2008). This procedure takes into account the uncertainty due to the estimated heterogeneity variance and the within-study variances, resulting in wider confidence interval estimates in analyses based on a small number of studies. As a result, confidence intervals may include 0 even when classical*z*-distribution confidence intervals would not. To assess the heterogeneity of trials, in each meta-analysis we used Cochrane’s*Q*-test to examine the null hypothesis that all studies estimated the same effect. We further computed*I*^2^to estimate the ratio of true heterogeneity to total observed variation.

Publication bias, or the tendency that studies with statistically significant results are more likely to find their way into the published literature, was examined by means of funnel plots, with Egger regression and trim-and-fill analysis for estimation of the adjusted effect size and of missing studies (Borenstein et al., 2009b). These procedures are based on the expectation that the publication bias will increase with a smaller sample size. In contrast, in the absence of publication bias, the sampling error is random, and the studies will be distributed symmetrically about the mean effect size or standard error, which can be verified in the funnel plot. The trim-and-fill procedure uses an iterative process to remove the most extreme small studies from the one side of the funnel plot, re-computing the effect size at each iteration until the funnel plot is symmetric about the (new) effect size. This trimming yields the adjusted effect size.

## 3 Results

### 3.1 Non-invasive stimulation characteristics

Table 1lists the studies and conditions included in the meta-analysis and their stimulation characteristics, split up into the two approaches discussed earlier—sustained stimulation and short interference.

**Table 1.** Non-invasive stimulation characteristics of the studies.

Study	Design (real - sham / target - ctrl) ^a^	Participants (real - sham / target - ctrl) ^a^	Age Mean ± SD (or Range)	tDCS / TMS	Target site	Reference / Control site or condition ^a^	Electrodes size / Coil type ^a^	Intensity / Frequency ^a^	Onset of task / Stimulation ^b^	Stimulation Duration
Sustained modulation of social and emotional processes										
Haihambo et al. (2024)	Between	22 - 19 (24%)	26.0 ± 3.0	Anodal tDCS-fMRI	Vermis (2 cm below inion)	Chin	5 * 7 cm	2.0 mA	20 min after stimulation	20 min
Ma et al. (2023) (Target / Control Task) ^c^	Between	25 - 27 / 28 - 26 (24%)	20.3 ± 3.0 - 20.7 ± 4.2 / 19.8 ± 2.3 - 20.5 ± 3.5	Anodal tDCS	Vermis (2 cm below inion)	Right upper arm	5 * 7 cm	2.0 mA	Concurrent with stimulation	20 min
Clausi et al. (2022) (Anodal / Cathodal) ^c^	Between	16 - 16 (69%)	25.7 ± 6.8 / 30.8 ± 11.5	Anodal / Cathodal tDCS	Vermis (2 cm below inion)	Right shoulder	6 * 7 cm	2.0 mA	35 min after stimulation	20 min
Oldrati et al. (2021)	Within	24 - 24 (25%)	22.5 ± 1.9	Anodal / Cathodal tDCS	Vermis (2 cm below inion)	Right cheek	5 * 5 cm	1.5 mA	Concurrent with stimulation	20 min
Ferrucci et al. (2012)	Within	21 - 21 (43%)	(20 - 49)	Anodal / Cathodal tDCS	Vermis (2 cm below inion)	Right shoulder	6 * 7 cm	2.0 mA	35 min after stimulation	20 min
Heleven et al. (2021)	Between	23 - 23 (30%)	24.6 ± 4.0	Repetitive TMS	Vermis (1-2 cm below inion)	Sham	Double cone coil	1,000 * 1 Hz	After stimulation	17 min
Schutter & Van Honk (2009)	Within	12 - 12 (0%)	(18 - 23)	Repetitive TMS	Vermis (1 cm below inion)	Sham, Occipital (3 cm above inion)	Iron core coil	1,200 * 1 Hz	After stimulation	20 min
Schutter et al. (2009)	Within	15 - 15 (0%)	20.4 ± 1.9	Repetitive TMS	Vermis (1 cm below inion)	Sham, Occipital (3 cm above inion)	Iron core coil	9,000 * 20 Hz	After stimulation	15 min
Short interference of social and emotional processes										
Ferrari, Ciricugno, Battelli, et al. (2022) (Exp. 1)	Within	32 - 32 (NR)	23.4 ± 1.8	Short pulse TMS	Vermis	Vertex, Left Cerebellum	Butterfly coil	3 * 20 Hz	At stimulus onset	---
Ferrari, Ciricugno, Battelli, et al. (2022) (Exp. 2)	Within	48 - 48 (NR)	23.5 ± 2.5	Short pulse TMS	Vermis	Vertex, Left Cerebellum	Butterfly coil	1 * 20 Hz	At or 100 ms after stimulus onset	---
Ferrari, Ciricugno, Battelli, et al. (2022) (Exp. 3)	Within	32 - 32 (NR)	23.5 ± 3.0	Short pulse TMS	Left Cerebellum	Vertex, Vermis	Butterfly coil	3 * 20 Hz	300 ms after stimulus onset	---
Ciricugno, Ferrari, et al. (2024) (Exp. 1)	Within	25 - 25 (28%)	23.9 ± 2.9	Short pulse TMS	Left paravermis	Vertex	Butterfly coil	3 * 20 Hz	20–120 ms after 2 ^nd^ stimulus	---
Ciricugno et al. (2024) (Exp. 2)	Within	24 - 24 (38%)	22.3 ± 2.4	Short pulse TMS	Left paravermis	Vertex	Butterfly coil	3 * 20 Hz	100–210 ms after 2 ^nd^ stimulus	---
Ferrari et al. (2023) (Exp. 1, Left)	Within	24 - 24 (17%)	22.8 ± 3.2	Short pulse TMS	Left cerebellum	Vertex	Butterfly coil	3 * 20 Hz	Before 2 ^nd^ stimulus	---
Ferrari et al. (2023) (Exp. 1, Vermis)	Within	24 - 24 (17%)	22.8 ± 3.2	Short pulse TMS	Left paravermis	Vertex	Butterfly coil	3 * 20 Hz	Before 2 ^nd^ stimulus	---
Ferrari et al. (2023) (Exp. 2, Left)	Within	22 - 22 (23%)	22.7 ± 2.2	Short pulse TMS	Left cerebellum	Vertex	Butterfly coil	3 * 20 Hz	Before 2 ^nd^ stimulus	---
Ferrari et al. (2023) (Exp. 2, Vermis)	Within	22 - 22 (23%)	22.7 ± 2.2	Short pulse TMS	Left paravermis	Vertex	Butterfly coil	3 * 20 Hz	Before 2 ^nd^ stimulus	---
Ferrari et al. (2023) (Exp. 3)	Within	28 - 28 (18%)	22.5 ± 3.2	Short pulse TMS	Left cerebellum	Vertex, Left paravermis	Butterfly coil	3 * 20 Hz	Before 2 ^nd^ stimulus	---
Ferrari et al. (2023) (Exp. 4)	Within	20 - 20 (15%)	21.9 ± 1.7	Short pulse TMS	Left paravermis	Vertex, Left Cerebellum	Butterfly coil	3 * 20 Hz	At stimulus onset	---
Ferrari, Ciricugno, Urgesi, et al. (2022) (Exp. 1)	Within	20 - 20 (55%)	23.2 ± 1.6	Short pulse TMS	Left cerebellum (Crus 1/2)	Vertex, Early Visual cortex	Butterfly coil	3 * 20 Hz	150 ms before 2 ^nd^ stimulus	---
Ferrari, Ciricugno, Urgesi, et al. (2022) (Exp. 2)	Between	20 - 20 (65%)	24.0 ± 4.6	Short pulse TMS	Left cerebellum (Crus 1/2)	Vertex, Early Visual cortex	Butterfly coil	3 * 20 Hz	150 ms before 2 ^nd^ stimulus	---
Ferrari, Oldrati, et al. (2018) (Exp. 1)	Between	18 - 18 (25%)	22.5 ± 2.2	Short pulse TMS	Left cerebellum	Vertex	Butterfly coil	3 * 20 Hz	150 ms before 2 ^nd^ stimulus	---
Ferrari et al. (2018) (Exp. 2)	Within	20 - 20 (35%)	23.7 ± 3.2	Short pulse TMS	Left cerebellum	Early Visual cortex	Butterfly coil	3 * 20 Hz	150 ms before 2 ^nd^ stimulus	---
Ferrari et al. (2018) (Exp. 3)	Within	20 - 20 (20%)	23.7 ± 2.6	Short pulse TMS	Left cerebellum	Early Visual cortex	Butterfly coil	3 * 20 Hz	150 ms before 2 ^nd^ stimulus	---
Gamond et al. (2017)	Within	20 - 20 (50%)	22.8 ± 0.8	Short pulse TMS	Right Cerebellum	Early Visual cortex	Butterfly coil	3 * 20 Hz	At stimulus onset	---

a Unless noted otherwise, headers before and after “/” refer to tDCS and TMS, respectively; for “Participants,” the % of males is indicated between parentheses; “Intensity” of all TMS studies was at 80% of the individual motor threshold for rTMS and at 100% for short pulse TMS; “Frequency” refers to the number of pulses for each stimulus; for short pulses this was either “1” (single pulse) or “3” (triple pulse).

b Onset of task for sustained modulation studies or stimulation in short interference studies.

c Participant and stimulation characteristics refer to condition before and after “/”.

NR, Not Reported; SD, Standard Deviation.

The sustained modulation studies (Table 1, top) involved both tDCS and TMS. The tDCS studies applied either anodal or cathodal stimulation, targeting the cerebellar vermis with electrode sponges of about 5 * 5 to 6 * 7 cm, using 1.5 to 2.0 mA for 20 min, before or during the task. Several return sites were used: cheek, chin, shoulder, or upper arm. The TMS studies applied repetitive TMS targeting the vermis, using different types of coils (a flat “double cone,” a slightly curved “double butterfly,” or “iron core”) using 1 to 20 Hz for 10 to 20 min, before the task. Several control conditions or sites were used (sham, occipital or early visual cortex, healthy control participants).

The short interference studies (Table 1, bottom) involved only TMS. These studies applied three short pulses of TMS (or one pulse in Experiment 2 ofFerrari, Ciricugno, Battelli, et al., 2022) targeting the vermis, left or right cerebellum, all using a slightly curved “butterfly cone” coil applying 20 Hz, either before, after, or at each onset of a target stimulus. The typical control site was the vertex, but also occasionally the vermis, left cerebellum, or early occipital visual cortex was used.

### 3.2 Stimulation outcomes

Table 2lists the outcomes of the studies. The dependent measures were either accuracy or response times. The Hedges’*g*effect sizes were coded in the direction of the hypothesis, that is, increasing performance for the sustained stimulation and increasing interferences for the short pulse TMS studies. The results of the pooled Hedges’*g*on each stimulation category are listed in the headers of each panel ofTable 2. Forest plots are depicted inFigure 3.

**Fig. 3.**
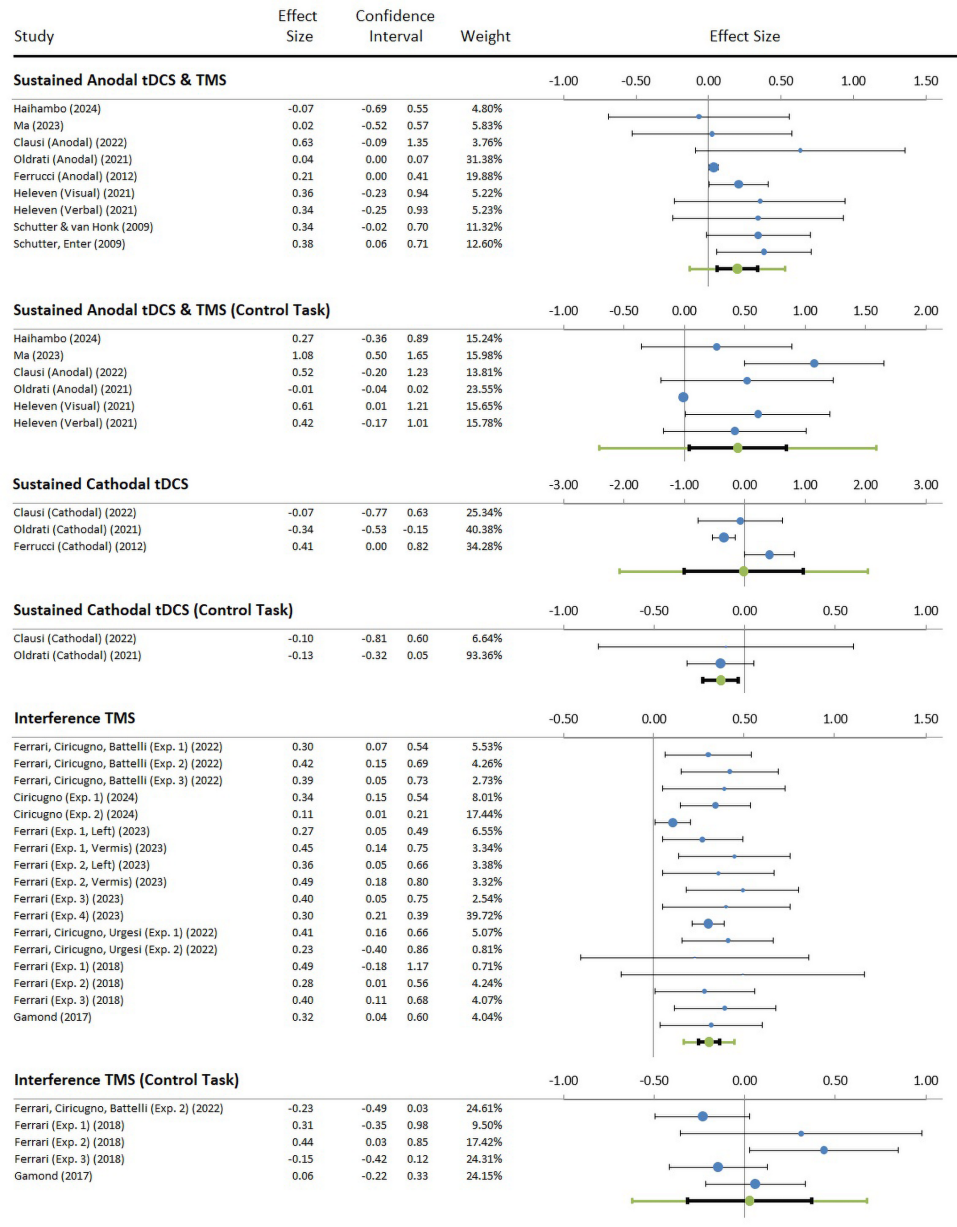
Forest plots depicting the effects of non-invasive transcranial stimulation. Dots represent each study, with dot size reflecting study weight in the model and error bars indicating the effect size (with confidence interval). The lower line in each plot represents the combined effect size (green dots) with its confidence interval (black narrow interval) and its 95% prediction interval (green wide interval). The latter gives the range in which, in 95% of the cases, the outcome of a future study will fall, assuming that the effect sizes of studies are normally distributed.

**Table 2.** Study outcomes in function of stimulation purpose and technique.

Study	Domain	Social or Emotional Target task	Non-social or Non-emotional Control task	RT (↑ faster; ↓ slower) Accuracy (↑ higher; ↓ lower) ^b^			Hedges’ *g* ^c^ (95% confidence interval)	
				DV	Target Task	Control Task	Target Task	Control Task
**Sustained modulation of social and emotional processes (Anodal tDCS / TMS)**							**0.20 (0.06 - 0.34)**	**0.44 (0.04 - 0.84)**
Haihambo et al. (2024)	Social Sequencing	Predicting the order of actions based on traits	Predicting the order of physical objects based on their characteristics	RT	=	↑	-0.07	0.27
Ma et al. (2023)	Social Sequencing	Implicit learning of the order of true & false beliefs	Implicit learning of the order of physical shapes	RT	=	↑ (total and orientation randomization)	0.02	1.08
Clausi et al. (2022) (Anodal)	Emotion - Social	Facial emotion & mental state discrimination	Gender discrimination / Visual discrimination	RT	↑	=	0.63	0.52
Oldrati et al. (2021) (Anodal)	Social Sequencing	Predicting social actions	Predicting movement of physical shapes	ACC	↑ moderately informative context	=	0.04	-0.01
Ferrucci et al. (2012) (Anodal)	Emotion	Facial emotion recognition	Visual Reorientation	RT	↑ negative emotions = positive emotions	=	0.21	NR
Heleven et al. (2021) (Visual)	Social Sequencing	Ordering actions involving true & false beliefs	Ordering physical events	RT	↑	↑	0.36	0.61
Heleven et al. (2021) (Verbal)	Social Sequencing	Ordering actions involving true & false beliefs	Ordering physical events	RT	↑	↑	0.34	0.42
Schutter & Van Honk (2009)	Emotion	Viewing or re-appraising negative emotional & neutral pictures	---	RT	↑ negative mood after exposure to pictures	---	0.34	---
Schutter et al. (2009)	Emotion	Emotional faces (implicit: masked after 14 ms and naming color of mask)	---	RT	↑ implicit response to positive pictures	---	0.38	---
**Sustained modulation of social and emotional processes (Cathodal tDCS)**							**-0.02 (-1.01 - 0.97)**	**-0.13 (-0.23 - -0.03)**
Clausi et al. (2022) (Cathodal)	Emotion— Social	Facial emotion & mental state discrimination	Gender discrimination / Visual discrimination	RT	=	=	-0.07	-0.10
Oldrati et al. (2021) (Cathodal)	Social Sequencing	Predicting social actions	Predicting movement of physical shapes	ACC	↓ strongly informative context	=	-0.34	-0.13
Ferrucci et al. (2012) (Cathodal)	Emotion	Facial emotion recognition	Visual Reorientation	RT	↑ negative emotions = positive emotions	=	0.41	NR
**Short interference of social and emotional processes (TMS)**							**0.31 (0.25 - 0.36)**	**0.03 (-0.32 - 0.37)**
Ferrari, Ciricugno, Battelli, et al. (2022) (Exp. 1)	Social Sequencing	Biological vs. scrambled moving light points discrimination	---	ACC	↓	---	0.30	---
Ferrari, Ciricugno, Battelli, et al. (2022) (Exp. 2)	Social Sequencing	Biological vs. scrambled moving light points discrimination	Inverted (upside down) movements	ACC	↓	=	0.42	-0.23
Ferrari, Ciricugno, Battelli, et al. (2022) (Exp. 3)	Social Sequencing	Biological vs. scrambled moving light points discrimination	---	ACC	↓	---	0.39	---
Ciricugno et al. (2024) (Exp. 1)	Emotion	Facial emotion discrimination	---	ACC	↓ at 120-220 ms	---	0.34	---
Ciricugno et al. (2024) (Exp. 2)	Emotion	Facial emotion discrimination	---	ACC	↓ at 100-210 ms	---	0.11	---
Ferrari et al. (2023) (Exp. 1, Left)	Emotion	Facial emotion discrimination	---	ACC	↓ discriminate between happy vs. angry faces	---	0.27	---
Ferrari et al. (2023) (Exp. 1, Vermis)	Emotion	Facial emotion discrimination	---	ACC	↓ discriminate between happy vs. angry faces	---	0.45	---
Ferrari et al. (2023) (Exp. 2, Left)	Emotion	Facial emotion discrimination	---	ACC	↓ discriminate between happy vs. fearful faces	---	0.36	---
Ferrari et al. (2023) (Exp. 2, Vermis)	Emotion	Facial emotion discrimination	---	ACC	↓ discriminate between happy vs. fearful faces	---	0.49	---
Ferrari et al. (2023) (Exp. 3)	Emotion	Situational context & facial emotion discrimination	---	ACC	↓ fear in congruent context	---	0.40	---
Ferrari et al. (2023) (Exp. 4)	Emotion	Single facial emotion recognition	---	ACC	↓ recognition of fearful faces	---	0.30	---
Ferrari, Ciricugno, Urgesi, et al. (2022) (Exp. 1)	Emotion	Bodily emotion discrimination	---	ACC	↓ discriminate between happy vs. angry body postures	---	0.41	---
Ferrari, Ciricugno, Urgesi, et al. (2022) (Exp. 2)	Emotion	Bodily (angry–sad and happy–surprise) emotion discrimination	---	ACC	↓ discriminate between sad vs. angry body postures; = happy-surprise	---	0.23	---
Ferrari et al. (2018) (Exp. 1)	Emotion	Facial emotion discrimination	Gender discrimination (implicit emotion discrimination)	ACC	↓ facial emotional discrimination	↓ implicit	0.49	0.31
Ferrari et al. (2018) (Exp. 2)	Emotion	Facial emotion discrimination	Gender discrimination (implicit emotion discrimination)	ACC	↓ facial emotional discrimination	↓ implicit	0.28	0.44
Ferrari et al. (2018) (Exp. 3)	Emotion	Facial emotion discrimination	Gender discrimination of neutral faces	ACC	↓ facial emotional discrimination	= (no emotions)	0.40	-0.15
Gamond et al. (2017)	Emotion	Ingroup bias (faster RTs to positive vs. negative words after ingroup face priming)	Outgroup bias	RT	↓ ingroup bias (faster RTs for negative words)	=	0.32	0.06

a Unless noted otherwise, headers before and after “/” refer to tDCS and TMS, respectively.

b ---, not measured; =, no significant effect.

c Effect size of significant effect(s) comparing in order of priority: active versus sham stimulation (always for tDCS and for TMS when noted), and if absent, target versus control site, or pre- versus post-stimulation. Note that the effect size is coded in the direction of the hypothesis, that is, facilitatory for sustain stimulation and inhibitory for short interference. Effect size for the control task that did not involve social or emotional inferences was provided when enough data were available. For each heading, the pooled Hedges’*g*is provided in bold for each task category with 0.95 confidence interval.

DV, main dependent variable; ACC, accuracy; RT, response time; NR, not reported.

#### 3.2.1 Sustained stimulation outcomes

While the anodal tDCS and TMS studies showed predominantly facilitatory and a few null effects, cathodal tDCS studies revealed mixed facilitatory and inhibitory effects. For a clear presentation, analysis, and conclusion, the sustained modulation studies were further subdivided into a top panel on anodal tDCS and TMS, and a middle panel on cathodal tDCS.

Sustained anodal tDCS or TMS shows a small significant pooled effect size on the social and emotional tasks of 0.20 (CI_95_0.06— 0.34,*z*= 3.26,*p*< 0.001) with effect sizes ranging from -0.07 to 0.63, while the control tasks reveal a significant moderate pooled effect size of 0.44 (CI_95_0.04— 0.84,*z*= 2.84,*p*= 0.002), with effect sizes ranging from -0.01 to 1.08. The pattern of effect sizes after anodal tDCS or TMS was homogeneous for the social and emotional tasks as revealed by a non-significant Cochrane’s*Q*statistic (*Q*= 15.33,*p*= 0.053;*I*^2^= 0.47), although this was heterogeneous for the control tasks (*Q*= 23.53,*p*< 0.001;*I*^2^= 0.79).

Typically, in most studies using anodal tDCS or TMS, effects were about equal on the target and control tasks, while tDCS was least effective in the study conducted byOldrati et al. (2021). A surprising finding is, however, that tDCS was ineffective on some social sequencing tasks, while it was much more effective on the non-social control tasks in two studies (Haihambo et al., 2024;Ma et al., 2023), with a small to large effect size of 0.27 and 1.08, respectively. The authors of these studies argued that this might be due to the higher familiarity of neurotypical participants with social tasks enabling them to perform already at ceiling even without stimulation, resulting in little improvement after stimulation, compared with non-social tasks which are often structurally identical, but less familiar so that progress is larger. We return to this issue in the discussion.

Cathodal tDCS shows a non-significant pooled effect size in the social and emotional tasks of -0.02 (CI_95_-1.01— 0.97,*z*= -0.07,*p*= 0.470) with a heterogeneous pattern (*Q*= 11.34,*p*= 0.003;*I^2^*= 0.82) of effect sizes ranging from -0.34 to 0.41. In contrast, in the control task, cathodal tDCS resulted in a significantly negative pooled effect size of -0.13 (CI_95_-0.23— -0.03,*z*= -0.17,*p*< 0.001), showing a limited range (*Q*= 0.01,*p*= 0.931;*I^2^*= 0.00) from an effect size of -0.10 to a -0.13.

#### 3.2.2 Short interference outcomes

The short TMS interference on the social and emotional tasks shows a small significant pooled effect size of 0.31 (CI_95_0.25— 0.36,*z*= 11.37,*p*< 0.001), with effect sizes revealing a limited range (*Q*= 21.00,*p*= 0.178;*I^2^*= 24%) from a small 0.11 to a moderate 0.49 effect size. Note that the effects are scored in the direction of the hypothesized stronger interference. The short TMS interference on the control tasks shows a negligible pooled effect size of 0.03 (CI_95_-0.32— 0.37,*z*= 0.22,*p*= 0.414), with effect sizes revealing a limited range (*p*= 1.00).

### 3.3 Sensitivity analysis of the pre–post correlation

To verify the impact of the imputed correlation of*r*= 0.80 between measures in the within-participant designs for which we did not have the exact data, we ran additional analyses with*r*from 0.70 to 0.90 in steps of 0.05. The effects size of all task categories remained very similar given this range of correlations, resulting in a change of Hedges’*g*and 95% confidence interval of ≤0.02, except for the sustained stimulation of socio-emotional tasks that, with a correlation of 0.90, showed a decrease of 0.03 for Hedges’*g*and 0.05 for the high confidence limit.

### 3.4 Publication bias

**Fig. 4.**
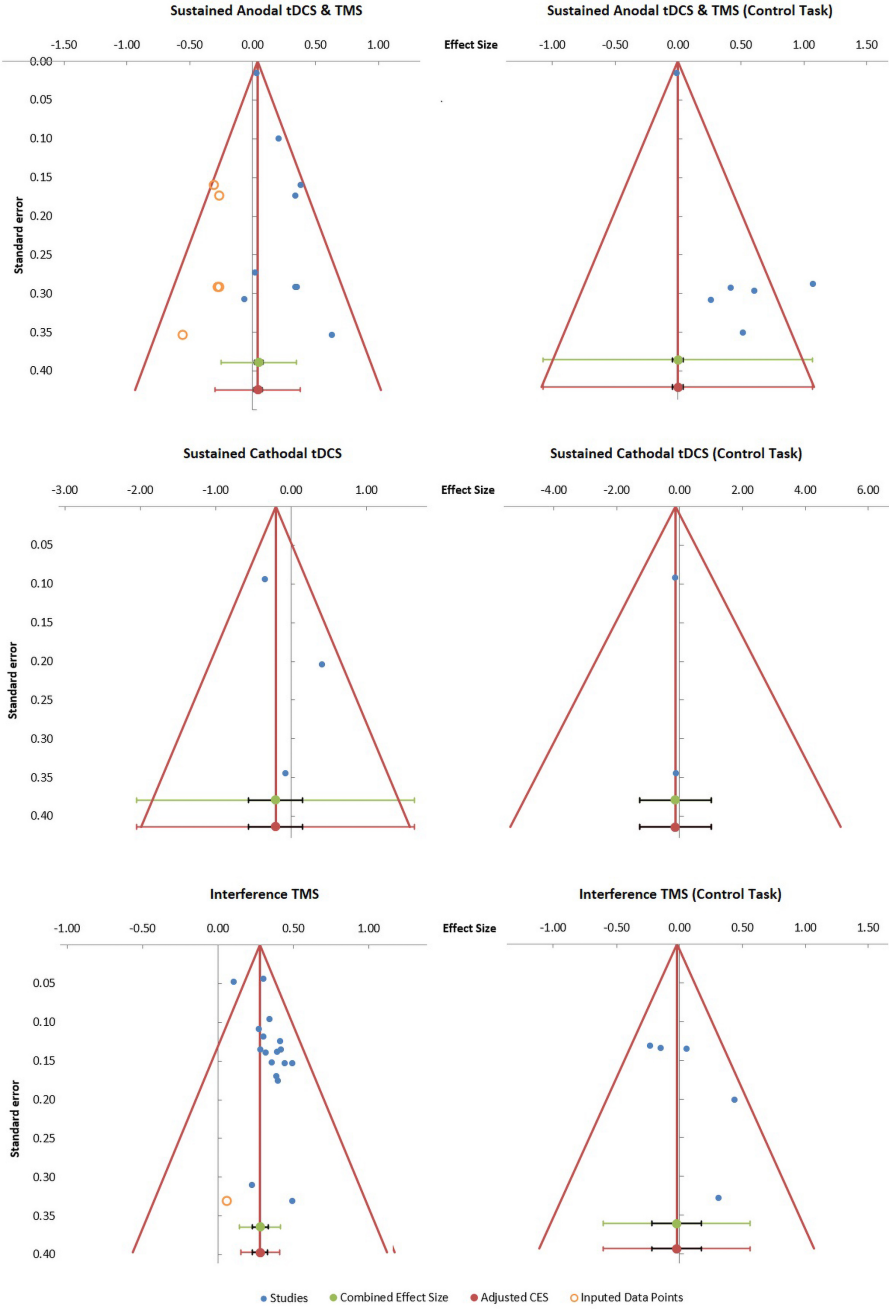
Funnel plots illustrating possible publication bias. Circular dots represent studies imputed by trim-and-fill analysis, the presence of which is evidence of asymmetry in the distribution of effect sizes and significant publication bias. The first lower line represents the combined effect size (green dots) with its confidence interval (black narrow interval) and its 95% prediction interval (green wide interval). The second lower line represents the adjusted combined effect size (CES) with its accompanying confidence interval (black narrow interval) and prediction interval (red wide interval), which represents the results of a trim-and-fill procedure after adjusting for missing studies (Duval & Tweedie, 2000a,2000b).

For the socio-emotional tasks, the funnel plot and trim-and-fill analysis (Fig. 4) were suggestive of a publication bias in the sustained modulation studies using anodal tDCS and TMS, with five missing negative studies and a significant Egger statistic (*t*= 3.33,*p*= 0.013), and in the interference studies using short TMS pulses, with one missing negative study and a significant Egger statistic (*t*= 2.96,*p*= 0.010). In contrast, no indication of a publication bias was found for the sustained modulation studies using cathodal tDCS, with zero missing studies and a non-significant Egger statistic (*t*= 0.96,*p*= 0.514).

For the control tasks, there was some indication of a publication bias in the sustained modulation studies using anodal tDCS and TMS, with zero missing studies but with a significant Egger statistic (*t*= 3.89,*p*= 0.018), and no publication bias in the interference studies using short TMS pulses, with zero missing negative studies and an non-significant Egger statistic (*t*= 1.70,*p*= 0.188). A publication bias could not be computed for the sustained modulation studies using cathodal tDCS due to lack of sufficient studies (only two).

In all studies with a suspected publication bias, the funnel plots suggested a substantive reduction of the effect size to zero, except for the short TMS interference studies which remained significant (Fig. 4).

## 4 Discussion

The present meta-analysis investigated the effects of non-invasive transcranial stimulation, specifically using tDCS and TMS, targeting the posterior cerebellum on the social and emotional understanding of others, also known as mentalizing or theory of mind. We found a growing number of studies in neurotypical samples using sustained modulation, where stimulation was applied before or during a social or emotional task, often with the aim of improving performance. The results provide strong evidence that sustained anodal tDCS and TMS generally improved social and emotional performance after stimulation, with a small effect size. This has important theoretical implications, suggesting that the cerebellum is causally involved in social and emotional cognition. In addition, these results may also have important clinical applications for pathologies in which social and emotional mentalizing is impaired (e.g., autism, schizophrenia). In contrast, cathodal stimulation showed mixed results.

In addition, there was converging evidence on the efficacy of short TMS pulses with the aim of interfering with ongoing social or emotional processes, with a small effect size. This approach is more theoretically oriented and, by significantly interfering with the processing at each social or emotional stimulus presentation, strongly supports a causal role of the cerebellum in socio-emotional cognition.

Our analysis included 14 studies comprising 19 socio-emotional and 13 non-socio-emotional stimulation conditions targeting the cerebellum. A summary of the main findings is listed inTable 3. The findings are subdivided in function of stimulation methodology, target site, and social or emotional measures. We discuss the results in more detail below separately for the sustained stimulation and short interference approaches. To explore the specificity of the transcranial stimulation effects, we analyzed the impact on socio-emotional mentalizing tasks in comparison with control (typically non-socio-emotional) tasks which did not require or allow mentalizing.

**Table 3.** Overview of performance after non-invasive tDCS or TMS.

tDCS / TMS	Domain	Target stimulation	Performance		
			Socio-emotional Improvement	Socio-emotional Impairment	Non-social ^a^ Improvement
**Sustained modulation**					
Anodal Tdcs	Social	Vermis	1		2
	Emotional		2		
Repetitive TMS	Social	Vermis	2		
	Emotional		2		
Cathodal tDCS	Social	Vermis		1	
	Emotional		1	1 ^b^	
Total			8	2	2
**Short interference**					
Short pulse TMS	Social	(Para)Vermis		3	
	Emotional			4	
	Social	Left Cerebellum		1	
	Emotional			8	
	Emotional	Right Cerebellum		1	
Total			0	17	0

Note: Entries are the number of studies showing significant change in performance. This review included 15 studies with 30 socio-emotional conditions. Social studies in this review include sequencing instructions or material (i.e., dynamic stimuli), including generating the correct order of actions involving social beliefs (Heleven et al., 2021), implicitly learning the order of social beliefs (Ma et al., 2023), discriminating biological moving light points (Ferrari, Ciricugno, Battelli, et al., 2022) and predicting the (order of) social actions (Haihambo et al., 2024;Oldrati et al., 2021). Studies on emotion understanding involved static visual presentations, including viewing motional pictures (Schutter & Van Honk, 2009), recognizing and discriminating facial emotional expressions (Ciricugno, Ferrari, et al., 2024;Clausi et al., 2022;Ferrari, Oldrati, et al., 2018;Ferrari et al., 2023;Ferrucci et al., 2012), implicitly recognizing emotional faces (Schutter et al., 2009), discriminating emotional body postures (Ferrari, Ciricugno, Battelli, et al., 2022;Ferrari, Ciricugno, Urgesi, et al., 2022), and implicitly recognizing emotional words after viewing ingroup faces, reflecting ingroup bias (Gamond et al., 2017).

a Sequencing of non-social objects only is improved (non-social effects are not listed when social condition is also improved).

b No change.

### 4.1 Sustained non-invasive stimulation

Sustained stimulation of the cerebellar vermis generally increased social and emotional task performance immediately after anodal tDCS and TMS stimulation, with a small but significant Hedges’ effect size of 0.20. Note that these effects are relatively optimistic, as in some studies that involved several task conditions, we selected only those that were significantly modulated by stimulation. Quite often, improvements were also found on the non-mentalizing control tasks in some of these studies, with a significant moderate effect size of 0.44. Effects on non-mentalizing control tasks based, for instance, on logical rules (Ma et al., 2023) are not surprising, given that the cognitive*executive control*cerebellar network is located just behind the cerebellar mentalizing network, which itself is positioned directly under the scalp (Buckner et al., 2011).

More surprising is the finding of improvement on non-social cognitive control tasks after tDCS, which can even last for 1 week, while no effect was observed on social tasks (Haihambo et al., 2024;Ma et al., 2023). One study showed small effect sizes on both social and control tasks (Oldrati et al., 2021). As noted earlier, a potential explanation is that social mentalizing is quite familiar and automatic for most neurotypical participants, so that they most likely perform this task at ceiling even before stimulation, resulting in little gain. In contrast, cognitive control tasks involving a similar presentation of objects and shapes are less familiar, even though they are structurally identical, leaving a larger margin for improvement after stimulation. For example, in the study ofMa et al. (2023), participants in the social condition had to infer very rapidly the true beliefs of smurfs (Belgian cartoon characters) when they were either looking at one or two flowers offered to them, or their false beliefs when they were oriented away from the flowers (and then participants had to report the number of flowers the smurfs falsely believed to have been given on a preceding trial). In contrast, in the non-social control condition, participants had to report the number of flowers on the screen or on a preceding trial (paralleling a true or false belief trial, respectively) on the basis of logical rules involving the combined presence of the shape (square or circle) and color of objects instead of smurfs.

This explanation is consistent with reports in the cognitive domain, which show stronger effects of cerebellar stimulation on difficult rather than easy tasks (e.g.,Pope & Miall, 2012) and other studies revealing a dependency of initial brain state on stimulation effects (Silvanto et al., 2008). This line of reasoning seems to be confirmed in the social domain, whereFerrari, Cattaneo, et al. (2018)found that TMS over the right cerebellum impaired the ability to recognize the correct order of appearance of novel geometric shapes while it did not affect the recognition of highly familiar short sequences of letters or numbers. Similarly, a patient study (De Vidovich et al., 2016) showed that patients with borderline personality disorder improved their responses to an emotion discrimination task after TMS, while neurotypical control participants did not. In autistic populations, recent meta-analyses of non-invasive transcranial stimulation revealed a significant reduction in autism symptoms after tDCS (Van Overwalle et al., in press) and TMS (Barahona-Corrêa et al., 2018;Smith et al., 2023) with larger Hedges’ effect sizes (between 0.41 and 0.58) than the present analysis. But note that these studies typically included only clinical scales rather than objective measures of social and emotional performance. Taken together, these findings suggest that the social or emotional (clinical) state or experience, prior to stimulation, is an important determinant of the potential gain and efficacy of non-invasive transcranial stimulation (Schutter et al., 2023), and this appears to be particularly true for highly familiar social and emotional processes. Note that although some studies report emotion-specific effects, in general, there is no selective preference for positive or negative emotional stimuli, asTable 2shows improvement or little change for both valences across studies. Likewise,Table 2shows that modulation of social and emotional processes was found across static and dynamic stimuli, lending little support for the idea that dynamic sequences of stimuli (Leggio & Molinari, 2015) might be more sensitive to non-invasive cerebellar stimulation. However, future studies using the same paradigm and stimulation methods, or directly comparing static versus dynamic stimuli, are needed to draw any definitive conclusions.

In conclusion, there is evidence that sustained cerebellar anodal tDCS and TMS effects are significant with small-to-moderate effect sizes, consistent with the general view that these techniques facilitate neural learning. Although three tDCS studies showed non-significant effects, the analysis revealed some suspicion of publication bias, which calls for more studies of cerebellar non-invasive stimulation in social and emotional cognition, and perhaps more importantly, for pre-registration of studies so that the level of null effects can be assessed.

In contrast, sustained cathodal cerebellar stimulation shows mixed results on both mentalizing and non-mentalizing tasks, with non-significant effect sizes of -0.02 and -0.13, respectively, consistent with the general finding in the literature that its effects are mixed and less well understood (Batsikadze et al., 2013;Klaus & Schutter, 2022;van Dun et al., 2017).

All sustained tDCS and TMS studies targeted the vermis (see summaryTable 3), so this seems an effective site for transcranial stimulation. However, little can be concluded from the present analysis on the best cerebellar site for stimulation.

### 4.2 Short interference

The analysis of short TMS pulses at stimulus presentation demonstrates that they are very effective in interfering with ongoing social and emotional functioning, as all studies reveal impaired performance across targeted cerebellar areas, with a pooled Hedges’ effect size of 0.34. There was no suspicion of publication bias, which attests to the robustness of the results. The effect on non-mentalizing control tasks was negligible. This points to the critical and preferential causal role of the posterior cerebellum in social and emotional cognition. However, research using short TMS pulses did not include sequential tasks except for one study (Ferrari, Ciricugno, Battelli, et al., 2022), so that the observed interferences effect does not contribute to studying the critical role of the cerebellum in sequencing. It is surprising that all other TMS interference studies present static photographs of emotional facial and bodily expressions. As noted earlier, non-invasive cerebellar transcranial random noise stimulation reduced accuracy to discriminate static sad facial expressions, but increased accuracy to discriminate dynamic sad facial expressions (Malatesta et al., 2024). Future research could benefit greatly from investigating dynamic emotional expressions or emotions embedded in a dynamic context, which predominate in real life and might demonstrate a greater contribution of the cerebellum. One study suggests that negative emotions are discriminated more accurately than positive emotions (Ferrari, Ciricugno, Urgesi, et al., 2022), but no strong conclusion should be drawn from this finding in the absence of further evidence.

Although summaryTable 3seems to suggest that stimulation site is less important in that both vermal and lateral cerebellar stimulation lead to interference, this is not entirely correct. For example, vermal TMS pulses impaired basic emotional discriminations, while lateral TMS pulses interfered with emotion recognition given a social context in which it was experienced, suggesting a vermal-to-lateral gradient of increasingly complex emotion identification (Ferrari et al., 2023).

### 4.3 Clinical implications and comparisons with stimulation of other brain areas

By facilitating neural excitability, sustained stimulation by anodal tDCS or TMS holds great potential for therapeutic use of transcranial stimulation in clinical populations who predominantly suffer from social and emotional difficulties. Non-invasive stimulation research in clinical populations is growing and has typically been applied to other cortical areas besides the cerebellum. We briefly illustrate this point with autism, because the most prominent diagnostic criterion of this disorder is difficulties in social interaction and mentalizing (American Psychiatric Association, 2013;Bylemans et al., 2023).

As mentioned earlier, targeting both cerebellar and cerebral areas, a recent meta-analyses of non-invasive transcranial stimulation on autistic samples (Van Overwalle et al., in press) reported significant reduction in autism symptoms and improvement in social performance after tDCS, with a moderate Hedges’ effect size of 0.58. Other recent meta-analyses reported a moderate TMS Hedges’ effect size of 0.41 (Barahona-Corrêa et al., 2018) and 0.44 (Smith et al., 2023) on the reduction of autistic symptoms. When targeting only social mentalizing areas in the cerebrum and cerebellum, the aforementioned analysis (Van Overwalle et al., in press) reported a moderate Hedges’ effect size of 0.54 for tDCS, and a negligible effect size of 0.02 for TMS (which involved only four experiments). This compares favorably with the present meta-analysis on neurotypical samples, where targeting cerebellar areas with sustained anodal tDCS and TMS shows a slightly smaller effect size of 0.27 for socio-emotional tasks and a moderate effect size of 0.44 for cognitive control tasks. It is unclear whether this weaker effect in the present meta-analysis is due to differences in samples (autistic vs. neurotypical) or types of measures (autistic symptoms vs. social and emotional tasks). As noted above, improved performance might be expected more in clinical populations for whom the social tasks are more difficult and are performed below ceiling prior to stimulation.

While the therapeutic potential in neurotypical and clinical populations is evident given the significant effect sizes ranging from small to moderate, the limited differences between cerebellar and cerebral targets seem to suggest that targeting different brain areas can improve mentalizing about others’ social and emotional behaviors. Targeting social brain areas may reinvigorate impaired social processes, whereas targeting cognitive executive areas (most commonly the lateral prefrontal cortex;Hyde et al., 2022) may enhance controlled strategies that may compensate for impaired mentalizing abilities.

One way to make progress in understanding how non-invasive transcranial stimulation modulates neural processes is the study of combined stimulation with neuroimaging, including functional magnetic resonance imaging (fMRI), functional near-infrared spectroscopy (fNIRS;Han et al., 2022), and electroencephalogram (EEG;Chan et al., 2023). fMRI studies are most suited for identifying the areas that are influenced by cerebellar stimulation. Recent fMRI studies found that non-invasive stimulation does not only modulate activity in the target cerebellar site, but also distal sites that are functionally connected. To illustrate, in the combined tDCS–fMRI study byHaihambo et al. (2024), cerebellar anodal tDCS was delivered before participants completed a social prediction task, which required them to correctly order randomly presented sentences describing actions of social agents, based on prior information on their personality traits. After tDCS, activation increased in mentalizing areas of the posterior cerebellum, including Crus 2 and lobule IX, as well as in remote mentalizing areas of the cerebrum, including the medial prefrontal cortex, temporo-parietal junction, and precuneus. Another concurrent fMRI-tDCS study targeting the vermis and right posterior cerebellum (Catoira et al., 2023) required participants to generate the correct temporal sequence of actions involving social beliefs, social routines, and non-social (control) events. The results revealed that this specific bi-cerebellar set-up decreased brain activation in mentalizing areas, including the temporo-parietal junction and the precuneus, and also resulted in decreased task performance. These two studies show that stimulation in critical cerebellar areas of a mentalizing network may have significant neural effects on the whole network encompassing both the cerebrum and cerebellum.

Yet another interesting approach is to use TMS for investigating the chronometry (i.e., timing) and causal connectivity of neural processes in the cerebellum. A recent chronometric TMS study (Ciricugno et al., 2024; see alsoSpampinato & Celnik, 2021) investigated the time course of posterior cerebellar activity in emotion discrimination by providing an inhibitory TMS pulse at different time points after the presentation of a facial emotional expression. The study revealed that the posterior cerebellum was recruited at an early stage of emotional processing (starting from 100 ms after stimulus onset), simultaneously with the posterior superior temporal sulcus (pSTS; a cortical area involved in human movement observation;Molenberghs et al., 2012;Van Overwalle & Baetens, 2009). To study causal connectivity, using a so-called condition-and-perturb TMS approach, this study also found that inhibitory TMS pulses over the cerebellum modulated the effects of TMS pulses delivered over the pSTS during emotion discrimination. This suggests that the activation of the pSTS during performance of a social task is dependent on cerebellar activation, providing evidence of the causal nature of their functional connectivity. Similar combined stimulation approaches have been used in motor and cognitive research (Silvanto et al., 2008;Spampinato & Celnik, 2021), while other studies explored the relative role of the cerebellum together with other brain areas in learning (Galea et al., 2011). These studies may provide inspiration for similar manipulations and designs to increase our insights on cerebellar chronometry and causal connectivity in social and emotional cognition.

### 4.4 Limitations

Given the significant effect sizes ranging from small to moderate on neurotypical populations, the existing pool of studies is too small to examine differences between the cerebellum versus the mentalizing or non-mentalizing cerebrum as stimulation targets, or to explore the differential effects on social and emotional behaviors with or without sequencing elements in more detail. Further technical protocol and montage differences such as intensity and length of stimulation, size, and exact position of target and return electrodes or specific TMS coils and so on are currently beyond the reach of any systematic analysis, given the limited number of studies with comparable simulation techniques, approaches, and samples. A cursory evaluation of the present data revealed little systematic differences given the location of the targeted area (lateral vs. vermal cerebellum), the size of tDCS electrodes (varying from 5 * 5 cm to 6 * 7 cm), the position of the tDCS return electrodes, or the shape of the TMS coil.

None of the studies in this analysis used double blinding, which would increase confidence in the obtained results. This should be an important focus for future studies. It is, therefore, encouraging to see that a very recent TMS study (outside the time window of our analysis) used double blinding (Ghezeljeh et al., 2024).

As noted earlier, the current meta-analysis suggests that stimulation of sequencing or non-sequencing material does not make much of a difference overall for sustained stimulation. Moreover, the great majority of short TMS interference studies did not include sequencing input. Therefore, the issue of whether transcranial stimulation can contribute to facilitating sequencing of socio-emotional actions is an important avenue for future exploration. The “*sequence detection hypothesis*” put forward byLeggio and Molinari (2015)and confirmed by a series of recent studies (Van Overwalle & Heleven, 2023;Van Overwalle et al., 2021) suggests that a primary role of the cerebellum is to identify the sequences of the events, which supports the accuracy, efficiency, and flexibility of social and emotional judgments. Future research may point out areas for further improvement of transcranial stimulation by stipulating which sequencing elements may lead to enhanced social and emotional cognition, or require more attention in therapeutic settings (e.g., for autism;Bylemans et al., 2022).

The short-pulse TMS studies were mainly conducted by a single team (involving Chiara Ferrari and Zaira Cattaneo (seeTable 2; bottom panel)), which may present a potential bias. This team of researchers use very similar stimulation protocols, including the same TMS coil, intensity, and frequency, which might be a strength in that the obtained effects are consistent and robust, devoid from methodological variations or inconsistencies. However, this seriously limits the generalizability of their findings to different types of cerebellar stimulation. The homogeneity in methodology and extensive coverage of the short-pulse TMS interference studies compared with the wider variety in the types of stimulation in the sustained tDCS and TMS studies also raises questions about how much weight can be put into the results of each type of stimulation. More variation in the protocols of inhibitory short-pulse TMS stimulation is needed to assess the generalizability of these findings, while more replications of sustained tDCS and TMS stimulation are needed to confirm the current (weaker) results.

## 5 Conclusion

The present meta-analysis showed significant evidence that sustained anodal tDCS and TMS generally improved social and emotional performance after stimulation compared with a sham or control condition, with a small effect size. However, there is a concern that this evidence is clouded by a publication bias. Control tasks without social or emotional component also showed significant improvement, suggesting that sustained stimulation may also affect other brain networks and functions. In contrast, cathodal tDCS showed mixed results. In addition, short TMS pulses aimed at disrupting ongoing social or emotional identification showed a small inhibitory effect size, which was highly specific, as this effect was absent in control tasks. Because this research is conducted by the same team of researchers, using the same protocols and designs, the generalizability of these findings might be somewhat limited. Together, these results show that cerebellar neurostimulation is effective in improving socio-emotional skills, supporting a causal role of the cerebellum on socio-emotional cognition, and may have important clinical applications in disorders with impairments in social and emotional mentalizing.

## Data Availability

The data from the meta-analysis are available from the first authors upon request.

## Author Contributions

Frank Van Overwalle conceived and conducted the research, ran the analyses, and wrote the draft manuscript. All authors extracted or checked the data in the studies involved, and edited the manuscript.

## Funding

This research was funded by the Strategic Research Program SRP57 from the Vrije Universiteit Brussel awarded to Frank Van Overwalle. Rocío Martínez-Regueiro is the beneficiary of a Margarita Salas Postdoctoral Fellowship co-funded by the Spanish Government and NextGen-EU funds. Mahyar Firouzi is a Fundamental Research fellow funded by the Research Foundation Flanders (Fonds Wetenschappelijk onderzoek, FWO), grant number 11G9622N.

## Declaration of Competing Interest

The authors have no competing interests to declare.
